# Sex‐specific aging in animals: Perspective and future directions

**DOI:** 10.1111/acel.13542

**Published:** 2022-01-23

**Authors:** Anne M. Bronikowski, Richard P. Meisel, Peggy R. Biga, James R. Walters, Judith E. Mank, Erica Larschan, Gerald S. Wilkinson, Nicole Valenzuela, Ashley Mae Conard, João Pedro de Magalhães, Jingyue (Ellie) Duan, Amy E. Elias, Tony Gamble, Rita M. Graze, Kristin E. Gribble, Jill A. Kreiling, Nicole C. Riddle

**Affiliations:** ^1^ Department of Ecology, Evolution, and Organismal Biology Iowa State University Ames Iowa USA; ^2^ Department of Biology and Biochemistry University of Houston Houston Texas USA; ^3^ Department of Biology The University of Alabama at Birmingham Birmingham Alabama USA; ^4^ Department of Ecology and Evolutionary Biology The University of Kansas Lawrence Kansas USA; ^5^ Department of Zoology University of British Columbia Vancouver British Columbia Canada; ^6^ Department of Bioscience University of Exeter Penryn UK; ^7^ Department of Molecular Biology, Cell Biology and Biochemistry Brown University Providence Rhode Island USA; ^8^ Department of Biology University of Maryland College Park Maryland USA; ^9^ Department of Computer Science Center for Computational and Molecular Biology Brown University Providence Rhode Island USA; ^10^ Integrative Genomics of Ageing Group Institute of Ageing and Chronic Disease University of Liverpool Liverpool UK; ^11^ Department of Animal Science Cornell University Ithaca New York USA; ^12^ Department of Biological Sciences Marquette University Milwaukee Wisconsin USA; ^13^ Milwaukee Public Museum Milwaukee Wisconsin USA; ^14^ Bell Museum of Natural History University of Minnesota Saint Paul Minnesota USA; ^15^ Department of Biological Sciences Auburn University Auburn Alabama USA; ^16^ Josephine Bay Paul Center for Comparative Molecular Biology and Evolution Marine Biological Laboratory Woods Hole Massachusetts USA

**Keywords:** aging, comparative biology, lifespan, mortality, sex differences

## Abstract

Sex differences in aging occur in many animal species, and they include sex differences in lifespan, in the onset and progression of age‐associated decline, and in physiological and molecular markers of aging. Sex differences in aging vary greatly across the animal kingdom. For example, there are species with longer‐lived females, species where males live longer, and species lacking sex differences in lifespan. The underlying causes of sex differences in aging remain mostly unknown. Currently, we do not understand the molecular drivers of sex differences in aging, or whether they are related to the accepted hallmarks or pillars of aging or linked to other well‐characterized processes. In particular, understanding the role of sex‐determination mechanisms and sex differences in aging is relatively understudied. Here, we take a comparative, interdisciplinary approach to explore various hypotheses about how sex differences in aging arise. We discuss genomic, morphological, and environmental differences between the sexes and how these relate to sex differences in aging. Finally, we present some suggestions for future research in this area and provide recommendations for promising experimental designs.

## Graphical abstract

Sex difference in aging occurs across the animal kingdom, but there is considerable variation and they are not universal. The processes leading to sex‐specific aging are poorly understood and might originate in sex‐specific genome architecture, organismal biology, or environmental interactions. Here, we take a comparative approach to review the various hypotheses and suggest promising areas of research for further study.

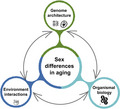

## 1 INTRODUCTION

Aging (or senescence) is the decline or deterioration in physiological, biochemical, and physical function seen with increased age. At the population scale, such deterioration in organismal phenotypes often manifests as declining fertility and increasing mortality with advancing adult age. It is well known that animal reproductive aging can differ between the sexes (reviewed in Comizzoli & Ottinger, 2021), with an extreme example being reproductive cessation in some female, but not male mammals (Alberts et al., 2013) with a long post‐reproductive lifespan being seen in human females (Levitis et al., 2013). Remarkably, sex differences in lifespan are observed in many animal species as well and can encompass differences between males and females in the age‐of‐onset of senescence, the rate of increase in age‐specific mortality, and/or the initial mortality rate in early adulthood—all of which can lead to sex‐specific aging trajectories. Although aging is a near universal phenomenon, sex differences in aging are varied throughout the animal kingdom, with some species showing extreme sex differences and others showing none (Austad & Fischer, 2016). For example, in the ant *Lasius niger*, female queens live up to 28 years, female workers live several years, and males typically die within 2–3 months (Jemielity et al., 2007). In contrast, species lacking sex differences in lifespan are well documented among a variety of different species groups, including many mammals (Austad & Fischer, 2016) and birds (Liker & Szekely, 2005). Currently, the causes of this diversity in sex differences in aging across the animal kingdom are not well understood and present a fascinating problem for comparative biologists.

To study sex differences in aging and lifespan requires an understanding of how senescence evolves, as well as its genetic and environmental components. The evolutionary genetics of senescence is the subject of ongoing investigation, particularly in wild‐dwelling populations where senescence evolved [reviewed in (Bronikowski & Promislow, 2005)]. Aging likely arises due to age‐specific mutation–selection balance. If selection declines with advancing age [a null expectation in populations that have young‐skewed age distributions (Hamilton, 1966), see Box 1], two genetic processes may occur. Mutations with deleterious late‐age phenotypes may accumulate across generations (Medawar, 1946) and/or antagonistically pleiotropic mutations may accumulate with positive fitness effects in early life and negative fitness effects in late life (Williams, 1957). If age‐specific selection differs between the sexes—for example, through sex‐specific sources and drivers of mortality, then sex‐specific senescence can evolve provided genetic variation exists. Such sexually dimorphic selection trajectories can arise through sex‐specific age distribution skews, sex‐specific responses to the environment, or sex‐specific habitats and behaviors (reviewed in Lemaître et al., 2020). As an extreme example, consider systems in which males compete for female mates with combat or bright ornamentation. It has been shown elsewhere that such behavioral and morphological differences between the sexes can increase male mortality relative to female mortality. Such a difference would give rise to different age structures between males and females, from which sex‐specific senescence can evolve (e.g., Kappeler, 2017; Schacht et al., 2017). A less extreme example is seen in sex differences in resistance to infectious disease, particularly during pregnancy when female mammals can be more susceptible to infection [as seen in Soay sheep (Leivesley et al., 2019)]. Here too, high mortality in females could give rise to sexual dimorphism in age structure, and concomitant sexual dimorphism in mutation‐selection balance.

**Age‐structured populations and selection**

**FIGURE 1:**
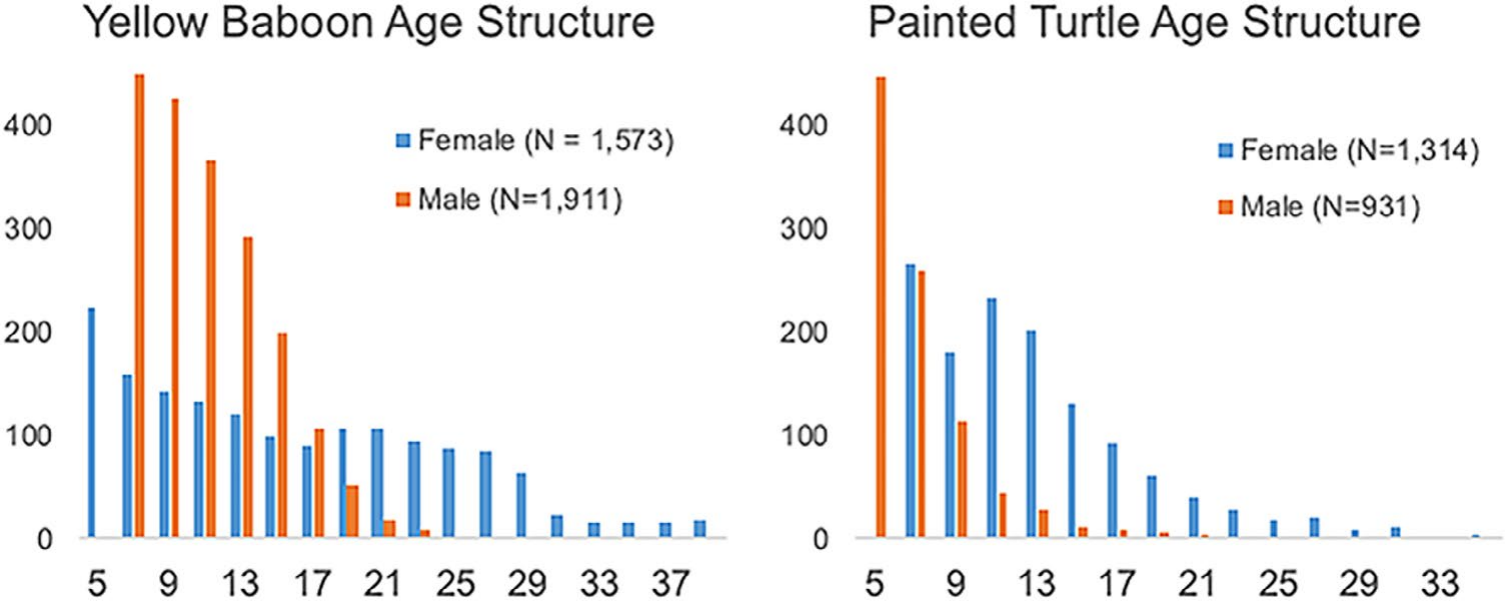
Sex‐specific age structure of adult wild baboons (*Papio cynocephalus*) and painted turtles (*Chrysemys picta*). Data from Bronikowski et al. 2011 and Bronikowski et al. 2021, respectively. In both populations, the male distributions are left‐skewed relative to the female distributions, and females have right extended distributions. The intensity of selection acting against a mutation that decreases age‐specific survival declines more rapidly with age for males in both species. Baboons have genotypic sex determination (degenerate sex chromosome in males). Painted turtles have environmental (temperature) sex determination (no sex chromosomes).

As Medawar noted (see text), senescence evolves in senescent‐free populations if age‐structure decreases with advancing age classes (for example, age‐independent predation or accidents that accumulate with number of years alive). This skew causes the strength of selection (i.e., the sensitivity of fitness to a small change in age‐specific survival) to decline with age (see Charlesworth, 1992). That is, the effect of a change in age‐specific survival early in life would have a much greater effect on population growth rate (*r* from the Euler‐Lotka equation) than an equal change later in life. The role of fertility is such that in species where fertility also declines with advancing age, the decline exacerbates the declining intensity of selection. Whereas, in species that exhibit increasing fertility with age (such as in turtles), an active area of study is whether such increasing fertility can offset the declines in selection intensity due to declining numbers of older individuals (See box 1 in Promislow and Bronikowski, 2006). The development of the formal mathematical theory for the evolution of senescence is attributed to Hamilton (1966). A full description of these considerations and theory can be found in Charlesworth (1992). Baboons and painted turtles were chosen to highlight the differences in sex‐determination mechanism (see Figure 1). Particularly interesting is the case of species with environmental sex determination, where genomic content and architecture are presumably identical between the sexes at fertilization.

Intimately entwined with sex‐specific selection is the mechanism of sex determination (Hägg & Jylhävä, 2021). Determining the evolutionary genetic dynamics and environmental contributors to age‐specific senescence from mechanisms of sex determination—sex chromosomes (heteromorphic, homomorphic, ancient, new, absent, etc.) and sex‐determining loci—remains a major challenge in the understanding of the evolution of sex‐specific aging. For example, an early driver of sexual differentiation once sex is “determined” is the reproductive steroid hormones (androgens and estrogens/progestins). Reproductive hormones are important in early sexual dimorphism in addition to their primary role in sexual maturation (reviewed in de Vries et al., 2014). But in oviparous TSD reptiles, maternally allocated steroid hormones in the yolk can also mediate sex determination itself (Bowden & Paitz, 2021). Disentangling the role of sex chromosomes, sex‐determining loci, and hormonally mediated sexual development is difficult, but there has been good progress in specific model organisms for this endeavor. For example, in the Four Core Genotype mice, these factors are decoupled, and work on this model has revealed potential independent contributions from both factors (Davis et al., 2019). Interestingly, nature has provided additional model organisms whose sex‐determination mechanism recommends them for addressing this question. For example, closely related species pairs that have genotypic sex determination versus environmental sex determination can reveal the role of sex‐specific genetic loci versus hormonal cascades in the development of sex‐specific aging (e.g., in reptiles, see turtle data in Box 1). At the mechanistic level, determining whether and which proximate (molecular) mechanisms of senescence have diverged between the sexes is a complementary challenge. To this end, we convened a series of workshops in October 2020, bringing together experts from a wide range of biological disciplines to tackle these questions. Here, we report on the ideas, questions, and challenges identified. Our emphases were genomic architecture differences between the sexes, including those deriving from sex‐determining mechanisms in contributing to sex‐specific aging and longevity.

### 1.1 Sex differences in aging in humans

Life expectancy of human females, on average, exceeds that of males across different human populations and historical times for which data are available (Mauvais‐Jarvis et al., 2020; Sampathkumar et al., 2020). This pattern is observed also in several other primate species (Bronikowski et al., 2011; Colchero et al., 2016). The longest recorded lifespan for women is 122 years, whereas for men it is 116 years. This difference in lifespan leads to marked female bias in the sex ratio of older cohorts. For example, the age pyramid of the United States shows the typical slight male bias in the youngest age group (0–4 years), then equivalency between males and females, until a bias toward females starts to appear at approximately 50 years of age (US Census Bureau). This female bias becomes most extreme in the oldest age group, 85 years or older, which includes more than twice as many women than men. This bias is also evident in the probability of reaching the age of 85, which is ~36% for men but ~50% for women based on the US Social Security Administration's Period Life Table for 2019. This shift with age toward an increased fraction of women among the surviving individuals is a common characteristic of human populations and illustrates how sex differences in aging impact population structure. By uncovering the mechanisms by which sex‐specific aging occurs in other organisms, we will better understand onset of disease and progression in the aging human population, which could include how we might reverse or slow this process.

In addition to the well‐documented sex differences in human lifespan, there are many other sex differences in how humans senesce (Austad, 2006; Austad & Fischer, 2016; Sampathkumar et al., 2020). These differences can be seen both at the organismal level as well as in various molecular measures. On the organismal level, while having an overall longer lifespan, women tend to be more “frail” and suffer more from physical ailments than men as they age (Collard et al., 2012; Gordon & Hubbard, 2020; Gordon et al., 2017; Marck et al., 2016). Osteoporosis, for example, is four times more common in women than in men. Women are also more prone than men to suffer from dementia and other age‐associated neurological diseases. For instance, women have approximately twice the risk of developing Alzheimer's disease than men (Ferretti et al., 2018). This increased level of frailty and physical impairment in women is partially due to their longer lifespan, but research suggests that other factors play a role as well, including genetics and possibly hormone biology (Ferretti et al., 2018). Despite this increased frailty seen in women, they die at lower rates than men from 13 of 15 top causes of death in the US (Austad & Fischer, 2016). On the molecular level, men and women show differences in how their immune response changes with age (Klein & Flanagan, 2016), and women tend to show fewer somatic mutations and chromosomal abnormalities than men with age [reviewed in (Fischer & Riddle, 2018)]. However, the molecular events that lead to the observed organismal level sex differences in human aging are not well understood.

### 1.2 Sex differences in aging across animal diversity

**FIGURE 2:**
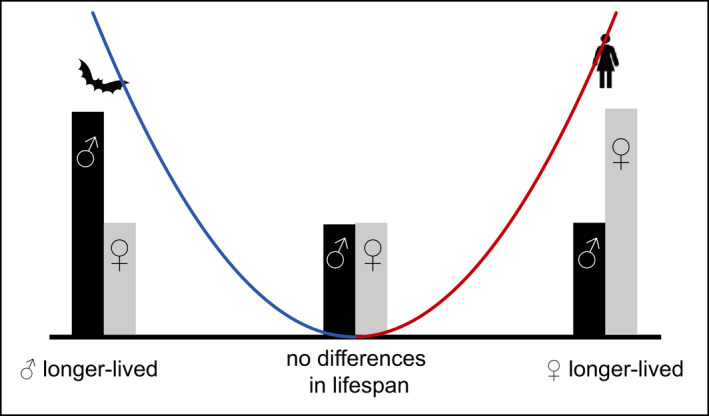
Sex differences in lifespan vary widely across animal taxa. Gray and black bars represent lifespan in females and males. Humans are an example of a species where females live longer, while in Brandt's bat, males live longer. See text for more examples. The curve illustrates that sex differences in lifespan (absolute value of lifespan(f)—lifespan(m)) form a continuum, from males living longer shown (left) to females living longer (right)

Like humans and many non‐human primates, a number of species exhibit female‐biased lifespans and slower aging (Austad & Fischer, 2016), yet numerous other species exhibit male‐biased lifespan and slower aging, and still others lack sex differences in aging entirely (Figure 2). Among mammals, females tend to have longer lifespans than males, but exceptions do exist. For example, lifespan is equivalent for both sexes in American beavers (Clutton‐Brock & Isvaran, 2007), and in some bat species, including Brandt's bat, males are longer lived than females (Kowalski et al., 2002; Podlutsky et al., 2005). In wild roe deer, which often show a female survival advantage, the increase in lifespan for females over males ranges from 0% to 30% depending on environmental factors (Garratt et al., 2015). This within‐class variation in longevity has been observed broadly across vertebrates, including birds, reptiles, amphibians, and fish (Tower & Arbeitman, 2009; Xirocostas et al., 2020). Similarly, insects and other invertebrates show striking variation in sex‐specific longevity. Social insects offer some of the most extreme examples, though are complicated by the fact that often two or more types of females exist with very different lifespans [i.e., worker and queen bees (Vaiserman, 2014; Xirocostas et al., 2020)]. Yet sex differences in aging are also known from other diverse insect taxa, including both hemimetabolous and holometabolous lineages (Bilde et al., 2009; Song et al., 2017; Zajitschek et al., 2009). Differences in lifespan between sexes or reproductive modes may be extreme in aquatic invertebrates as well; females of the rotifer *Brachionus manjavacas* live twice as long as males, and fertilized sexual females live 25% longer than asexual females (Snell, 2014). Sex differences in lifespan also vary widely in dioecious and androdioecious nematode worms, and again, outcomes seem to vary substantially depending on rearing conditions (Ancell & Pires‐daSilva, 2017). These examples illustrate that sex differences in terms of lifespan are widespread and highly variable across the animal tree of life.

Other aspects of senescence also show sex differences in diverse animal taxa. For example, reproductive potential declines with age at different rates in males and females of many species (Comizzoli & Ottinger, 2021; Holmes et al., 2001). In the red wolf, *Canis rufus*, male reproductive success, as measured by pup recruitment, declines with age, while no such decline is observed in females (Sparkman et al., 2017). This situation is reversed in the black‐footed ferret (*Mustela nigripes*) with females showing decreasing fertility after 3 years of age, while male fertility drops later, at 6–7 years of age (Wolf et al., 2000). Further, on the molecular level, we see that sexes can differ in the rate of decrease of telomere length (Barrett & Richardson, 2011; Gardner et al., 2014). In some species, males with a shorter lifespan have shorter telomeres earlier in life than females, as is the case in humans and many laboratory rodents (Barrett & Richardson, 2011; Gardner et al., 2014). There are also species such as ants and gulls where there is neither a relationship between telomere length and lifespan nor a sex difference in the telomere decay rate (Fischer & Riddle, 2018). For example, in two species of long‐lived bats, no relationship between telomere length and age was detected in either sex (Foley et al., 2020; Lorenzini et al., 2009; Power et al., 2021), and a recent meta‐analysis of 51 taxa found no consistent sex differences in telomere length (Remot et al., 2020). The examples of reproductive senescence and telomere length degradation illustrate the tremendous variation across animal taxa in the organismal and molecular features that show sex differences with aging.

### 1.3 Mechanisms leading to sex differences in aging

In 2013, Lopez‐Otin and colleagues proposed nine “hallmarks of aging,” that is, features that are commonly seen in aging animals across a wide range of species (López‐Otín et al., 2013; for a similar characterization of seven “pillars of aging” see Kennedy et al., 2014). These shared characteristics of aging include “genomic instability, telomere attrition, epigenetic alterations, loss of proteostasis, deregulated nutrient‐sensing, mitochondrial dysfunction, cellular senescence, stem cell exhaustion, and altered intercellular communication” (López‐Otín et al., 2013, see Box 2). The relationships among these molecular hallmarks, and their relationship to senescence—either causal or consequential—remain unknown outside of a few model species (i.e., humans, mouse, fruit flies).

**Molecular mechanisms of aging**

**Genomic instability:**

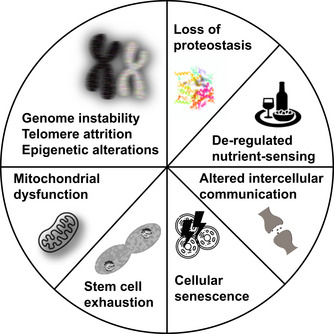
Increasing genome instability with age (i.e., rates of aneuploidy, somatic mutations, and dysregulation of transposable elements [TEs]) is well documented in many species. Evidence for a clear causal relationship to senescence is largely lacking, but data from model species suggest that improved DNA repair and increases in TE silencing can lead to longer lifespans. In addition, in humans, premature aging syndromes are linked to DNA repair and genome instability.

**Telomere attrition:**

Telomere attrition in humans correlates with age, and telomere attrition is directly related to cellular senescence (a primary cause of inflammation) and altered gene expression in sub‐telomeric regions (Dong et al., 2021). In mammals, the level of telomere maintenance with age depends on body size, with telomerase activity negatively correlated with body mass (Tian et al., 2018). Thus, there are a significant number of species documented, which lack telomere attrition, but nonetheless show physiological aging similar to species that show telomere attrition.

**Epigenetic alterations:**

Epigenetic changes with aging are widespread and include changes to cytosine methylation (Wilkinson et al., 2021), histone modifications, and chromatin structure. Heterochromatin loss associated with aging occurs in several species and improved maintenance of heterochromatin extends health‐ and lifespan (Ngian et al., 2021; Wood et al., 2016). In addition, a recent study demonstrated that expressing the key reprogramming genes *Oct4*, *Sox2*, and *Klf4* in old mice resulted in the re‐establishment of “young” cytosine methylation patterns and improved physiological functions (Lu et al., 2020), suggesting that epigenetic change underlies senescence.

**Loss of proteostasis:**

To maintain cellular proteins in a functional state, protein production, folding, modification, and degradation have to be carefully balanced. This balance is lost with advancing age, leading to a variety of problems. A causal link to aging is supported by studies that report increased lifespan in animals overexpressing chaperone proteins that promote proteostasis (Bobkova et al., 2015; Hsu, Murphy & Kenyon, 2003; Morley & Morimoto, 2004; Morrow et al., 2004; Yokoyama et al., 2002).

**Deregulated nutrient‐sensing:**

Nutrient‐sensing pathways in animals include the IIS (Insulin and Insulin‐like growth factor [IGF‐1] signaling), sirtuin, AMPK (AMP kinase), and mTOR (mechanistic target of Rapamycin) pathways. These pathways are responsible for assessing the body's nutritional needs and status, and their ability to do so decreases with age. Studies from several model species suggest that modulating these pathways genetically or by dietary restriction can impact life‐ and healthspan.

**Mitochondrial dysfunction:**

Mitochondrial function tends to decline with age. At the same time, mtDNA mutations increase with age. The combined roles of mitochondrial function, oxidative stress, mutational load, and mitochondrial mass have been the subjects of decades of research. Notwithstanding, the precise mechanisms by which mitochondrial phenotypes contribute to senescence remain relatively unknown.

**Cellular senescence:**

Loss of proliferative capacity is the main feature of cellular senescence (Di Micco et al., 2021). The accumulation of senescent cells leads to chronic localized inflammation. Senescent cells tend to have high levels of p16^ink4a^, which inhibits cyclin‐dependent kinases. Removal of cells with high p16 levels delays age‐associated disorders in mice (Baker et al., 2011; Che et al., 2020). However, this removal also has negative consequences and does not always lead to the desired senescence‐delaying effects (Grosse et al., 2020).

**Stem cell exhaustion:**

Stem cell exhaustion refers to the shrinking pool of stem cells with age (Ren et al., 2017). Over time, stem cells lose their capacity to produce differentiating daughter cells while maintaining their stem cell properties. Currently, it is mostly unclear to which extent stem cell exhaustion contributes to aging in general.

**Altered intercellular communication:**

With increased age, intercellular communications change and affect endocrine and neuronal communication between cells (see López‐Otín et al., 2013) and references therein). Particularly impacted are immune functions, with inflammatory reactions tending to increase with age, while surveillance against pathogens and malignant cells decrease (Borgoni et al. 2021). The overall contribution, causal or correlative, of altered cell‐to‐cell communication (beyond inflammaging (Franceschi et al., 2018) is not clear (but see Yousefzadeh et al., 2021 for a promising transplant study).

Research has revealed a variety of pathways contributing to aging, as summarized in the discussion of the nine hallmarks of aging (López‐Otín et al., 2013; but see Gems & de Magalhães, 2021). The molecular basis of sex differences in some species and absence of these differences in other species is not well defined, currently. Sex differences in any of the molecular drivers leading to the hallmarks of aging, or other biological mechanisms absent from the hallmarks, could potentially be involved. For example, males and females might have different levels of DNA repair enzymes, leading to different efficiencies in DNA damage repair and genome instability, thus impacting aging. This might be the case in Drosophila, as overexpression of DNA repair genes can impact males and females differently (Shaposhnikov et al., 2015). Similarly, males and females might differ in the rate at which they produce or clear senescent cells, thus leading to different rates at which senescent cells accumulate in various tissues. Sex differences in aging could also involve tissue‐specific pathophysiological mechanisms affecting specific organs, including sex‐specific organs, and resulting in age‐related pathologies (for an example of this in *Drosophila*, see Regan et al., 2016). While some data from model organisms exist for some of the molecular drivers (Fischer & Riddle, 2018; Menees et al., 2021; Mitchell et al., 2016; Rodriguez‐Fernandez et al., 2020; Tsurumi & Li, 2020), comprehensive investigations across taxa are lacking. Thus, the molecular pathways involved in generating sex differences in aging could be diverse and are poorly understood.

While sex differences in some molecular pathways associated with aging have been documented (reviewed in Hägg & Jylhävä, 2021; see also Baar et al., 2016; Brown‐Borg et al., 1996; Hwangbo et al., 2004; Selman et al., 2008; Svensson et al., 2011; Yoon et al., 1990) the triggers of these differences remain obscure (but see Chen et al., 2012; Sawala & Gould, 2017; Spaziani & Radicioni, 2020). Ultimately, sex‐specific selection (potentially arising from sexual selection) likely drives these sex differences in aging hallmarks, and the triggers of sex differences in aging are closely coupled with sexual differentiation pathways. Males and females often differ in (i) genome composition (e.g., sex chromosomes); (ii) adult morphology and life‐history (e.g., adult sexual size dimorphism, reproductive investment); and (iii) environments—both biological environments (e.g., hormonal milieu, microbiomes, parasites), and ecological environments (e.g., partitioning of home ranges / breeding grounds, intra‐ and inter‐sexual competition). How these biological and ecological differences between the sexes interact and impact the molecular drivers of aging and thus precipitate sex differences in aging is an important open question in the comparative biology of aging. Here, we summarize hypotheses and data related to genomic, morphological, and environmental differences between the sexes and how these relate to differences in senescence and lifespan. We end by suggesting future research emphases in this area.

## 2 GENOMIC DIFFERENCES BETWEEN THE SEXES AND DIFFERENCES IN AGING

Genomic differences between the sexes can arise at fertilization. Thus, sex‐specific aging may derive, in part, from differences in genotypes between males and females related to sex‐determination mechanisms.

### 2.1 Species with sex chromosomes

In species with sex chromosomes, the genomes of the two sexes differ in their sex chromosome complement. While other systems exist, most sex chromosomes generally fit into one of two categories: XX/XY systems, where males are the heterogametic sex (XY genotype) and females are homogametic (XX); and ZZ/ZW systems, where females are heterogametic (ZW) and males are homogametic (ZZ). There is considerable variation within these two sex chromosome types, as they have evolved multiple times independently (Bachtrog et al., 2014). Despite this variation, there is broad, empirical support for shorter lifespans in the heterogametic sex compared to the homogametic sex (Xirocostas et al., 2020), although with much variation (Marais et al., 2018). An analysis focused on tetrapods suggested that the sex‐determination system (XY vs. ZW) explained ¼ to ⅓ of the differences in the adult sex ratio (a proxy of sex differences in adult mortality) observed between species, depending on the heterogametic sex (Pipoly et al., 2015). A similar result was obtained in a study focused on amphibians (Cayuela et al., 2021). Data from the four core genotypes in mouse (animals with either XX or XY sex chromosome complement and either ovaries or testes generated by exploiting a translocation of the SRY gene to an autosome) shows that the presence of two X chromosomes improves lifespan, irrespective of the gonads (Davis et al., 2019). Together, the available data suggest that sex chromosomes might have a role in generating sex differences in aging (Marais et al., 2018).

Several potential explanations for the shorter lifespan of the heterogametic sex have been proposed. First, the “unguarded X” (or “Z”) hypothesis (Trivers, 1985) suggests that the heterogametic sex (i.e., with only one full sex chromosome X in males of XY species, and Z in females of ZW species) might express more deleterious morphological and physiological characteristics. This prediction derives from the observation that recessive deleterious X‐ or Z‐linked alleles are likely to be masked by a dominant beneficial allele in the homogametic sex, but are exposed to selection in the heterogametic sex. Despite solid theoretical foundations, the unguarded X hypothesis has mixed empirical support (Brengdahl et al., 2018; Sultanova et al., 2018, 2020). Second, the “toxic Y” (or “W”) hypothesis (Brown et al., 2020; Marais et al., 2018; Nguyen & Bachtrog, 2020) suggests that the Y or W chromosomes (which are sex‐limited) might account for sex differences in lifespan. Y‐linked genetic variation for male lifespan exists in *D*. *melanogaster* (Griffin et al., 2015) and chinook salmon (McKinney et al., 2020). Consistent with the role of the Y chromosome in aging, repetitive DNA on the Y chromosome is de‐repressed in older *Drosophila melanogaster* males, leading to mis‐expression of Y chromosome repeats as a function of aging. Data from humans also support the presence of a toxic Y effect, as the presence of an additional Y chromosome in males (i.e., XYY) leads to a 10‐year reduction in lifespan (Stochholm et al., 2010). In contrast, human males with an additional X chromosome (XXY, Klinefelter) only show a 2‐year reduction in lifespan (Bojesen et al., 2004). Thus, either the “unguarded X” or “toxic Y” hypotheses may help explain the shorter lifespan of the heterogametic sex, but the relative importance in various species is unknown.

X (or Z) chromosome dosage compensation may also have sex‐specific effects on lifespan—either by upregulation of the single sex chromosome in the heterogametic sex or by downregulation of the duplicate sex chromosome in the homogametic sex. Some taxa have evolved sex‐specific regulation of the X or Z chromosome in the heterogametic sex, which is predicted to compensate for the haploid expression of X‐ or Z‐linked genes (Ohno, 1967). In *Drosophila*, the X chromosome is upregulated in hemizygous XY males to equilibrate with the diploid dosage of the autosomes (Lucchesi & Kuroda, 2015). Misregulation of dosage compensation in males could explain shorter lifespan of *Drosophila* males. In comparison, in humans, where one copy of the X chromosome is inactivated in XX females (with some important exceptions, Tukiainen et al., 2017), biased (non‐random) inactivation of one copy of the X over the other is associated with shorter lifespan (Chuaire‐Noack et al., 2014; Gentilini et al., 2012; Ostan et al., 2016). However, as this process occurs in females, it does not explain the shorter male lifespan. Thus, while sex‐specific gene regulation via dosage compensation might impact sex differences in aging in some species, we maintain that it is not a general mechanism underlying sex differences in aging across taxa because of the idiosyncratic nature of dosage compensation across eukaryotes (Gu & Walters, 2017; Mank, 2013).

### 2.2 Species with haplodiploidy

Species where sex is determined by the presence/absence of chromosome pairs provide another opportunity to investigate the possible impacts of genomic differences between males and females on sex differences in aging. Species with haplodiploidy are best known among hymenopteran insects (e.g., bees, ants, wasps; female diploid, male haploid), but also include some scale insects and rotifers (Blackmon et al., 2017). In addition, some insects have sex differences in the elimination of the paternally inherited chromosomes, which can result in effective haploidy of the somatic genome (Gardner & Ross, 2014). Social Hymenoptera are of particular interest in terms of sex differences in aging, as often the sexes differ significantly in terms of lifespan, but also because there often are multiple classes of females—extremely long‐lived queens and sterile workers with lifespan more similar to the males. In these species, something similar to the “unguarded X” hypothesis might explain the sex differences in aging, as essentially the entire chromosome complement is unguarded in males, leading to the phenotypic manifestation of any deleterious alleles present (Smith & Shaw, 1980). However, this hypothesis does not explain the extensive lifespan differences between castes of one sex, such as the diploid sterile workers and the diploid fertile queens, suggesting that other aspects of these animals’ biology (e.g., gene expression, environments, diet) precipitate these lifespan differences.

### 2.3 The mother's curse: female‐specific inheritance of mitochondrial genomes

An additional genetic difference between the sexes involves the inheritance of mitochondria, which typically are passed from mother to offspring. The matrilineal inheritance of mitochondria means that selection on mitochondrial genomes (mtDNA) occurs only in females, leading to the prediction that males may suffer a “mother's curse” from mtDNA alleles that are optimized for female‐specific needs with respect to energy metabolism (Gemmell et al., 2004; Innocenti et al., 2011). Evidence for the mother's curse in animals is mixed (Dowling & Adrian, 2019), but it has been hypothesized to explain shorter lifespans in males than females (Camus et al., 2012; Frank & Hurst, 1996). For example, a study of Leber's hereditary optical neuropathy, a condition caused by mitochondrial mutations, discovered that these mutations lead to worse phenotypic outcomes in males than in females and are maintained in the population due to the matrilineal mtDNA transmission (Milot et al., 2017). The extent of the mother's curse may further depend on temperature for poikilotherms (Montooth et al., 2019), and we discuss how differences in temperature and energy metabolism can lead to sex differences in aging below. Moreover, the male‐limited inheritance of the Y chromosome may result in male‐specific adaptations that negate the mother's curse or even create a countervailing “father's curse” on autosomal loci (Ågren et al., 2019). The compounding effects of these sexually antagonistic selection pressures with sex‐biased modes of inheritance could lead to sex differences in aging. Nevertheless, it is difficult to predict which sex should outlive the other without knowing the values of many different parameters in a variety of population genetics models. For this reason, the mother's and father's curses remain intriguing explanations for sex differences in aging, but are of limited predictive value.

## 3 PHENOTYPIC DIFFERENCES BETWEEN THE SEXES AND DIFFERENCES IN AGING

In many species, in addition to the difference in sex organs, males and females also differ in a variety of features with varying degrees of sexual dimorphism. Extreme sexual dimorphism is often associated with mating systems. When males physically compete for access to females, the males tend to be the larger sex and may develop weaponry such as horns or antlers (e.g., Bro‐Jørgensen, 2007; Lindenfors et al., 2007; but note that in ~70% of bovids, females also have horns: Lundrigan, 1996). When females choose mates among males, male ornamentation in plumage, horns, vocalizations, or bright colors often occurs (Zuk & Simmons, 2018). In contrast, females may evolve ornaments in response to sexual selection or other selection pressures (Murray et al., 2018; Tobias et al., 2012). These ornaments can be costly to develop or maintain and thus may contribute to sex differences in aging (reviewed in Tidière et al., 2015). In addition, sperm competition might also be costly for males, thus impacting sex differences in aging (Lemaître et al., 2020). Sexual dimorphism also extends to a variety of morphological, physiological, and likely, molecular characteristics. In humans, for example, males and females differ in how their muscle develops and changes with age (Gheller et al., 2016). Experimental evolution studies on wheel‐running in mice have resulted in sex‐specific differences in lifespan, morphology, and physiology. For example, males are heavier than females in both high‐running and control mice, but both sexes of the high‐running strains are smaller than the control animals and the two sexes also show differences in body composition and corticosterone levels (Bronikowski et al., 2006; Castro et al., 2021). Given the wide‐ranging phenotypes that show sexual dimorphism, they need to be considered as potential causes of sex differences in aging (Tobias et al., 2012).

### 3.1 Sexual dimorphism in body size

Sexual size dimorphism is common in many groups. In mammals, males are often larger than females, whereas in reptiles (including birds) and insects, females are often larger than males. Ray‐finned fish exhibit the widest range of sexual size dimorphism across animals, with variation from dwarf and parasitic males to males that are more than 12 times larger than females (Fairbairn, 2015). Factors influencing the extent and direction of sexual dimorphism in body size include competition for mates and resources, mating systems, predation risk, and diet. For example, selection for increased fecundity often favors large females (e.g., reptiles: Bronikowski & Arnold, 1999), while extensive competition among males for access to females favors large males (e.g., mammals: Andersson, 1994; Weckerly, 1998), and selection for a shorter time to sexual maturity can lead to smaller animals of both sexes.

Differences in selective pressures between females and males, and the resulting sexual dimorphisms in size, must be considered in the context of life‐history strategies. For example, along the fast‐to‐slow pace‐of‐life continuum, suites of life‐history traits undergo correlated evolution toward more fast‐paced (fast growth, shorter lifespan) or slow‐paced (slow growth, longer lifespan) (e.g., Gangloff et al., 2020; reviewed in Dammhahn et al., 2018]. Selective pressures that cause the evolution of slower‐ or faster‐paced life histories can differ between the sexes and result in significant variation in morphological dimorphism and lifespan (Fairbairn et al., 2007; Maklakov & Lummaa, 2013). The brown antechinus (*A. stuartii*), a small marsupial, provides an extreme example, as all males die after mating, while ~10%–15% of females survive multiple mating seasons (Fisher & Blomberg, 2011). Phylogenetic constraints in sexual size dimorphism appear to be mostly absent; within many clades, sexual size dimorphism ranges from non‐existent, to males being twice as large as females, or females being much larger than males (Bronikowski et al., 2011; Ceballos et al., 2013; Cheverud et al., 1985; Ota et al., 2010; Rohner et al., 2018). In at least some species groups (birds and mammals; Promislow, 1992; Promislow et al., 1992), sexual dimorphisms and sexual selection are linked to sex differences in aging (but see Lemaître et al., 2020; Tidière et al., 2015).

Sexual dimorphism in body size is relevant to sex differences in aging as there are several connections between body size and aging. Generally speaking, among species, larger animals live longer than smaller animals (Speakman, 2005), whereas within species, the opposite can be true [e.g., dwarf mice (Bartke & Brown‐Borg, 2004; de Magalhães & Faragher, 2008) or dogs (Fleming et al., 2011; Kraus et al., 2013)]. This relationship between body size and lifespan is likely due to the fact that the pathways controlling lifespan and body size overlap at least partially. For example, dwarf mice are deficient for growth hormone, which leads to their small body size. However, the growth hormone deficiency also leads to increased insulin sensitivity and impacts insulin signaling (reviewed in List et al., 2021), which is an important modulator of lifespan. Interestingly, the lifespan extension seen in mice for growth hormone deficient animals is not seen in humans with growth hormone deficiencies (Bartke, 2021), suggesting that the link between growth regulation and lifespan is complex.

The allometric scaling of lifespan, that is, smaller animals living longer within species, however, is unlikely to explain sex differences in aging. For example, in primates, males tend to be larger than females, which leads to the prediction that males should live longer based on their body size, which is not normally seen. In addition, the environment, especially diet, also has significant impacts on body size and aging. Caloric restriction (CR)—within limits—often leads to increased lifespan, while high calorie diets lead to accelerated aging. Indeed, CR is known to extend lifespan in a sex‐specific and strain‐specific manner, which is well documented in mice and fruit flies (reviewed in Garratt, 2020 and Krittika & Yadav, 2019). In some insects, CR results in smaller, longer‐lived animals. In the short‐lived killifish *Nothobranchius furzeri*, CR extends lifespan of males, but not females (McKay et al., 2021). In *Caenorhabditis elegans*, CR increases lifespan in hermaphrodites, but does not impact the lifespan in males (Honjoh et al., 2017). These examples illustrate the complex nature of the body size/lifespan relationship and how sexual dimorphism in body size might contribute to sex differences in aging.

### 3.2 Sexual dimorphisms related to differences in developmental timing

**FIGURE 3:**
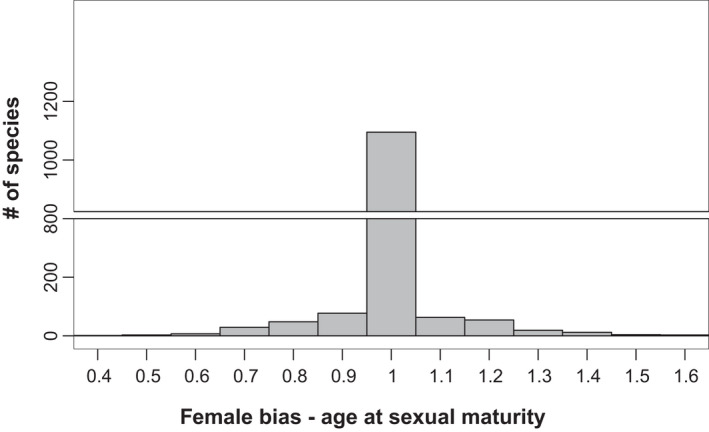
Variability in female age bias at sexual maturity across chordates. Female age bias is defined as female maturation age divided by the mean maturation age of both sexes. This distribution is centered at 1 (i.e., no age bias), with range from 0.42 to 1.62, with equal tails. Data from AnAge (birds contribute 48% of the data, mammals 40%)

In addition, one needs to consider the potential impact of differences in growth patterns and developmental timing on sexual dimorphisms that may result in sex differences in aging. While many species have genetically programmed growth cessation (i.e., determinate growth), species with indeterminate growth lack this limit and can theoretically continue to grow when resources are available and the environment permits. While male and female animals both begin development from a single identically sized cell, once developmental programs for each sex have been initiated, the two sexes have the potential to develop at different rates as they respond to different hormonal or environmental cues. This process can lead to large differences between females and males in age of maturity (Figure 3; de Magalhães et al., 2005), as well as sex differences in body size and other traits.

Sex differences in growth patterns are particularly common in poikilotherms (e.g., insects, fishes, and reptiles), and are of interest in aging biology because indeterminate growth can lead to indeterminate fecundity, which can change selection pressures in adult animals dramatically (Promislow & Bronikowski, 2006). In insects, the impact of growth rate and sex differences in developmental timing on body size is illustrated well by a study of black scavenger flies (Rohner et al., 2016), which focused on populations of *Sepsis neocynipsea* in North America (males larger than females) and Europe (females larger than males). While in most insects, females are the larger sex, Rohner and colleagues report growth rate differences between the sexes as well as a role for prolonged male development in the populations that show larger male body size.

In fish, Pacific salmon species (*Oncorhynchus* spp.) that are anadromous and semelparous exhibit varying degrees of sexual dimorphism and rapid senescence after spawning. Sexually mature sockeye salmon (*O*. *nerka*) are sexually dimorphic where males are generally larger, with deeper bodies and longer jaws than females (Johnson et al., 2006; Quinn, 2018; Quinn & Foote, 1994). Growth rates vary between anadromous salmon as they can spend 1–4 years feeding and growing to their final adult size in the ocean before returning to their spawning sites (Dittman & Quinn, 1996; Quinn, 2018), creating size‐age variation at spawning and subsequent death. In contrast, in Atlantic salmon (*Salmo salar*), 1%–6% of females survive after spawning, and return to the ocean where they can recover and spawn again (National Research Council et al., 2004; Tessier & Bernatchez, 2000). Thus, some Atlantic salmon are iteroparous, creating a unique population of older females.

In reptiles, plasticity in growth rates can be greater in one sex than the other (e.g., turtles: Ceballos et al., 2013, 2014; Ceballos & Valenzuela, 2011), reflecting sex‐specific selective pressures that can impact life histories, including aging (e.g., Bronikowski et al., 2021; Hoekstra et al., 2018). These examples of sex‐specific growth and development illustrate how sex‐specific selection pressures can give rise to (or result from) these patterns. In turn, variation between the sexes in selection and generation time may give rise to variation in strategies of somatic maintenance between the sexes, and the molecular mechanisms that underlie such maintenance (see Box 2). While this variation between the sexes might impact sex differences in aging, it can be difficult to disentangle the effects of sex‐specific growth rate, differences in absolute age at sexual maturity, and differences in body size. Likely, careful manipulations in model species will be needed, with the goal of altering one trait while keeping the others constant (for example, see Lind et al., 2017 for a study in *C. elegans*).

## 4 ENVIRONMENTAL DIFFERENCES BETWEEN THE SEXES AND DIFFERENCES IN AGING

### 4.1 Sex‐specific responses to the environment

The environment experienced by the two sexes also needs to be considered as a potential factor impacting sex differences in aging. Due to genetic differences and sexual dimorphism in various phenotypes, males and females might experience and respond to the environment differently, which, in turn, can influence sex‐specific trajectories of mortality and the molecular mechanisms underlying these trajectories. Temperature is a well‐studied environmental variable with sex‐specific responses. In general, at higher temperatures, animals have to expend more energy to maintain proteostasis as more chaperones, including heat shock proteins, are required to ensure proper protein folding (Somero, 2020). This gives rise to thermal critical maxima (and minima) that are species‐ and sex‐specific (reviewed in Bodensteiner et al., 2021). These effects of heat on cellular and biochemical functions may explain why ectotherms live longer at colder temperatures (Conti, 2008; Keil et al., 2015; Miquel et al., 1976).

Heat stress differentially impacts males and females in several species. In *Drosophila melanogaster*, male fertility is impacted more significantly than female fertility at higher temperatures (Zwoinska et al., 2020). In reptiles, differential effects of temperature on growth and immune function in the two sexes have been reported in common garden experiments (e.g., Palacios et al., 2020), including effects on survival (Addis et al., 2017). Addis and colleagues report for garter snakes that the impact of temperature depends on sex as well as ecotypes, with one ecotype (lower elevation, fast growth) showing increased female mortality at low temperatures, which is absent in males and both sexes of the second ecotype (higher elevation, slow growth; Addis et al., 2017). Similarly, male fertility is negatively impacted by high temperatures in many mammals, often with a lesser effect seen in females (Takahashi, 2012). Given the importance of energy allocation toward reproduction versus somatic maintenance for the progression of aging (Kirkwood, 1977), these examples illustrate how temperature might impact sex differences in aging.

Studies in seabirds, snakes, and beetles further illustrate how other environmental effects can influence sex differences in aging. A study from a seabird, the imperial shag *Leucocarbo atriceps*, revealed a complex interaction between fledgling weight, resource availability, and social environment (sibling number) (Svagelj et al., 2021). Males are typically larger than females, and in poor years, chicks of both sexes without siblings showed worse performance. In good years, male chicks weighed less in the presence of a sibling, while female fledgling weight was unaffected by the social environment (Svagelj et al., 2021). In great skua (*Stercorarius skua*), females are larger than males, and female chicks typically grow faster than males, but poor environmental conditions led to slower growth in female chicks than in male chicks (Kalmbach et al., 2009). Sex‐specific effects of development under poor nutrition also have been studied in garter snakes (Holden et al. 2019) where females, but not males, had significantly lower adult survival when they developed under poor nutrition (despite exhibiting catch‐up growth when switched to a high‐nutrient diet pre‐maturation). In humans, intra‐uterine growth restriction leads to negative outcomes more often in males (reviewed in Cheong et al., 2016). These findings demonstrate that identical environments can have very different impacts on the two sexes, leading to suboptimal outcomes in one sex, but not the other, potentially impacting survival and aging.

A sex‐specific environmental effect on lifespan has been explicitly demonstrated, for example, in the seed beetle *Callosobruchus maculatus* (Sanghvi et al., 2021). Sanghvi and colleagues manipulated larval density to determine the impact of early life environment quality on flight performance, fecundity, and lifespan. They found that female fecundity and lifespan are negatively affected by poor larval environment, while male fecundity and lifespan are not affected (Sanghvi et al., 2021). Another example is a study of Seychelles warbler, *Acrocephalus sechellensis*, which demonstrates that the presence of non‐breeding or co‐breeding helper females increased survival for older dominant females significantly, but did not do so for dominant males. Interestingly, dominant females lacking helpers showed increased rates of telomere attrition compared to females with helpers, while male telomere attrition rate was not impacted by the presence of helpers (Hammers et al., 2019). These two studies demonstrate that identical environments can have very different impacts on survival and aging for males and females and hint at possible mechanisms. However, assigning causal relationships in these kinds of studies can be difficult as even simple manipulations can have a variety of effects and impact various molecular pathways linked to aging.

Environmental factors also contribute to sequential hermaphroditism in fishes, where there are three patterns of sex change: (1) protogynous (female‐to‐male), (2) protandrous (male‐to‐female), and (3) serial bidirectional (Avise & Mank, 2009; Edgecombe et al., 2021). All patterns of functional sex reversal include restructuring of the gonad plus changes in behavior and morphology, which can include body size (Godwin, 2009; Nakamura et al., 2015; Warner, 1984). These data show that in addition to morphology and behavior, biological sex can be plastic. How this impacts age‐specific trajectories of mortality and lifespan is unknown. A dominant theory explaining the adaptive significance of the timing, direction, or pattern of sex change is the size advantage model, which details how sex change is adaptive when the reproductive value is greater when one sex is small and the other sex is older and thus larger (Ghiselin, 1969; Kazancioğlu & Alonzo, 2010; Munday et al., 2006; Warner, 1975). The timing of sex change should therefore maximize expected lifetime reproductive success (Warner, 1988). Sex‐specific size advantages associated with different mating systems will drive the direction of hermaphroditism within a species (Munday et al., 2006), and thus environmental factors affecting size attainment can directly influence sex change and alter individual lifespan. These sex‐specific responses to the environment potentially impact aging.

### 4.2 Sex‐specific environments

In addition to sex‐specific responses to shared environments, sex‐specific aging can occur if the environments experienced by the two sexes differ, which can begin in utero in mammals and viviparous reptiles, or at oviposition if mothers alter egg contents in an offspring‐sex‐dependent manner. Similarly, differences in preferences between the sexes for habitat, thermal profiles, and diet can also result in sex‐specific environments. Such sex‐specific environments are the norm for many species, often due to sex‐dependent dispersal behavior. Many mammals form matrilineal social groups with males dispersing among groups, which results in males and females experiencing very different social environments as subadults (e.g., primates: Bronikowski et al., 2011). Indeed, such male dispersal is seen in most polygynous mammal species (Clutton‐Brock & Lukas, 2012). As another example, most temperate bat species form female‐only “maternity colonies'’ with males living elsewhere. Even though both sexes are migratory, only females return to these maternity colonies (Senior et al., 2005). While female‐biased dispersal was thought to be typical of socially monogamous birds (Greenwood, 1980), phylogenetic analysis has not confirmed an association between mating system and sex‐biased dispersal (Mabry et al., 2013).

In addition to sex‐specific environments due to dispersal behavior, competition environments may also differ between the sexes. In many polygynous mammals, such as elephant seals, males aggressively compete with other males for access to mates while females avoid such dangerous conflicts and typically spend time obtaining sufficient resources for rearing offspring (reviewed in Fairbairn, 2015). Such competition among males can be energetically costly and cause injury and death. Mathematical modeling suggests that this sex‐specific “live fast, die young” strategy can evolve under conditions with short mating seasons and intense competition among individuals of one sex, which results in increased investment into reproductive effort coupled with minimal investment into post‐mating somatic maintenance (Fisher et al., 2013). While this type of reproductive strategy is rare in mammals, it is widespread in other taxa, such as many fishes, where differences in the competitive environments experienced by males and females lead to different life‐history strategies and sex differences in lifespan and aging. It also should be noted that these sex‐specific life‐history strategies do not only result in sex‐specific environments, but are linked also to the evolution of size dimorphisms and other traits that might impact aging independently.

Interactions between the two sexes and parental care represent other aspects of a sex‐specific environment with the potential to impact sex differences in aging (Klug et al., 2013). In many species, mating itself (irrespective of successful fertilization) can impact lifespan. In *Drosophila melanogaster*, male lifespan is reduced by mating and by even just the perception of the opposite sex (Gendron et al., 2014). Likewise, females living in the presence of males have shorter lifespan than expected when accounting for egg production (Harvanek et al., 2017; Landis et al., 2021; Partridge & Farquhar, 1981; Partridge et al., 1987), but a recent study found surprisingly small effects of mating on lifespan across 15 Drosophila strains (Hoffman et al., 2021). In many species, parental care is strongly dimorphic (reviewed in Clutton‐Brock & Scott, 1991). For example, in many mammals, females will provide for their offspring, first in utero, later nursing their young, and eventually training them to provide for themselves. The contribution from the male parent ranges from sperm‐only to the extended participation in child rearing by both parents seen in humans. Sexually dimorphic parental care is also seen in archosaurs (birds and crocodilians) and cichlid fishes (reviewed in Gans, 1996; Reynolds et al., 2002). Although most reptiles do not show extensive parental care per se, females and not males often engage in nesting behaviors (such as digging nests in oviparous species, and cessation of foraging in viviparous species: reviewed in Gans, 1996; Reynolds et al., 2002). A study of 37 Western Palearctic bird species found parental care to significantly impact sex differences in lifespan, while size dimorphisms did not (Owens & Bennett, 1994). Given the potential for sex differences in energy investment in reproduction, offspring survival, and parental care, this energetic dimorphism can lead to a sex difference in somatic maintenance and, ultimately, senescence and lifespan.

### 4.3 Species experiencing a range of environments

The previous two sections illustrate that both physical (e.g., temperature, resource availability) and behavioral (reproduction, competition) environments can impact sex differences in aging and lifespan. However, there are interesting intraspecific polymorphisms that provide evidence that sex differences in aging are robust across diverse physical environments (e.g., temperature, altitude, seasonality) and behavioral variation, although the magnitude of these sex differences can change. Some marine turtles, for example, have wide ranges that span from North America to Australia. Interestingly, similar sex‐specific aging trajectories in loggerhead turtles occur in populations that differ substantially in their ages of sexual maturity (20 years in N. America vs. 35 years in Australia; Mayne et al., 2020]. In contrast, one population of painted turtles along the Mississippi River exhibits sex‐specific lifespans and aging rates, whereas other populations do not (Congdon et al., 2003; Reinke et al., 2020). Additionally, garter snakes with populations of fast‐ or slow‐aging snakes (at low and high altitude, respectively) show greater skew in male vs. female reproductive success in the fast‐aging populations and exhibit sex‐specific effects of a SNP in mitochondrial Cytochrome B on metabolic rate and aging/lifespan (Gangloff et al., 2020). Mexican cave fish (*Astyanax mexicanus*) are a particularly fascinating example of a species with a sex‐by‐habitat interaction in the evolution of metabolic traits (Riddle et al., 2021), but without sex‐specific or habitat‐specific lifespan (Riddle et al., 2018). Migratory species are also of interest, as there can be migratory and non‐migratory populations that experience vastly different environments and stresses, but still show similar levels of sex differences in aging and lifespan. A recent comparative study of birds and mammals showed that migrant species and populations tended to have a faster‐paced life‐history strategy relative to non‐migratory species (Soriano‐Redondo et al., 2020). A study of Chesapeake Bay striped bass demonstrated that migrations tended to be undertaken only once animals achieved a specific size/age and that the non‐migratory animals experienced higher mortality than migratory animals. Interestingly, the sex distribution of migratory animals was skewed toward females, but it is unclear if this is due to differential mortality or aging between the sexes or sexual dimorphism in body size (Secor et al., 2020). These examples illustrate how studying species experiencing diverse environments can provide insights into sex differences in aging.

Finally, short‐lived species where subsequent generations experience different environmental conditions are informative. Insects that have spring versus winter generations might have different phenotypic morphs, but still experience sex differences in aging and lifespan. For example, *Drosophila suzukii* overwinter as adults, and females will lay eggs after the cold period, and these winter morphs show significantly longer lifespans than summer morphs (Shearer et al., 2016). In the desert locust, the gregarious morph has a shorter lifespan than the solitary morph. In monarch butterflies, summertime “reproductive” individuals showed far more pronounced sex differences in aging than autumnal migratory individuals in reproductive diapause (Herman & Tatar, 2001). These examples illustrate that, for some species, intrinsic factors might be more important than extrinsic environmental factors in generating sex differences in aging, but that for other species, the interaction between intrinsic (biological) and extrinsic (environmental) factors might be key.

## 5 OPPORTUNITIES FOR RESEARCH INTO SEX DIFFERENCES IN AGING

As the examples in the previous sections illustrate, there is immense variation among male and female animals in chromosome complement, morphology, and life history, as well as in the contribution of environment to sex differences in aging. In this section, we focus on genomic differences between the sexes as an especially promising avenue to understand sex differences in longevity and aging. Our premise is that careful selection of species can reveal how genome content and dynamics can contribute to sex‐specific aging in both inter‐ and intra‐species experimental designs. Below, we highlight three particularly promising areas related to genome differences between males and females: sex chromosomes, genome instability, and gene regulatory cascades.

### 5.1 Sex chromosomes in sex‐specific aging

As highlighted in Section 2, genomic differences between the sexes can arise at fertilization due to sex chromosomes. Sex differences in aging that may arise from sex chromosomes are likely due to the differences in gene content between the X and Y (or Z and W). In some animal groups, including mammals, *Drosophila*, and caenorhabditid worms, the X and Y (or Z and W) are highly differentiated, or heteromorphic. The Y or W has fewer functional genes and more repetitive elements compared to the X (or Z) (Charlesworth & Charlesworth, 2000; Graves & Marshall Graves, 2006; Rice, 1996). However, sex chromosome differentiation occurs across a continuum, and numerous animal species lack heteromorphic sex chromosomes having instead poorly differentiated, or homomorphic, sex chromosomes, which still retain many of their functional genes and lack significant increase in repetitive elements.

Two competing hypotheses for sex‐specific aging are the unguarded X/Z (more genes on the larger X/Z chromosome reveal unmasked deleterious alleles in the heterogametic sex), and the toxic Y/W (more repetitive sequence on the Y/W are deleterious in the heterogametic sex) (see Section 2). In the first case, comparison across species with an X (or Z) with more genes, for example, on a larger chromosome, would predict a greater longevity differential between males and females due to the number of genes impacted by hemizygosity. We might also predict a similar effect in haplodiploid species where the entire genome is effectively unguarded in the haploid sex, with all deleterious alleles being unmasked. The greatest challenge with haplodiploid species is that sexual dimorphisms in life‐history strategies often overwhelm lifespan differences, but solitary Hymenoptera could be promising models that overcome this challenge. Under the unguarded X/Z hypothesis, we would also predict little to no sex differences in aging in species that lack heteromorphic sex chromosomes (e.g., homomorphic or lack of sex chromosomes altogether, such as species with environmental sex determination). Comparisons among closely related taxa with and without heteromorphic sex chromosomes would resolve this question. Alternatively, under the toxic Y/W hypothesis, comparison across species with a Y or W chromosome ranging in size from smaller to larger predicts a greater sex differential in aging in species with larger sex‐specific chromosomes (i.e., Y or W). Similarly, we predict no observable effect in XX/XO or ZO/ZZ species where the Y or W has been lost entirely (Cochran & Ross, 1967; Fraïsse et al., 2017; Voelker & Kojima, 1971). Data from two *Drosophila* species illustrate this approach. Comparing *D*. *pseudoobscura* and *D*. *miranda*, both species with XY sex determination, but with Y chromosomes of different evolutionary age and size (*D*. *p*.‐ old, smaller; *D*. *m*.‐ young, larger), *D*. *miranda* shows more expression and transpositions of deleterious Y‐linked transposable elements (Wei et al., 2020), illustrating how a size‐dependent toxic Y effect might operate.

One limitation of inter‐species comparisons is that there are confounding differences among species other than sex chromosomes that may also affect aging. Thus, a complementary approach to comparing different species is to examine the effect of Y‐linked variation in aging within species that have documented Y polymorphisms (i.e., SNPs, copy number variation in repeats, structural variants). For example, Y chromosome haplotypes in *D*. *melanogaster* have *trans* effects on gene expression and chromatin throughout the genome in both XY males and XXY females (Lemos et al., 2008, 2010). Females do not transcribe any Y‐linked genes, and therefore, the effect of the Y chromosome on female transcriptional regulation is most likely caused by the chromatin content of the Y chromosome. Comparing aging in males and females carrying different Y chromosome types would allow a direct test of the effect of different Y chromosomes. Other *Drosophila* species have documented Y chromosome polymorphisms (Archer et al., 2017; Branco et al., 2013; Dobzhansky, 1937), which offers the exciting possibility of performing both within and among species comparisons in this model genus.

### 5.2 Genome instability in sex differences in aging

Following from the above hypotheses on the direct role of sex chromosomes on sex‐specific aging, there may exist genome‐wide phenomena that indirectly derive from sex determination. Animal species range in genome differentiation from heteromorphic sex chromosomes to species with haplodiploidy, where females have twice the genomic content of males, to environmental sex determination, where genome content is identical between the sexes. Of particular interest is closely related species that show different levels of genome differentiation or different modes of sex determination. In these species, it might be possible to untangle the impact of genomic differences on sex differences in aging from other factors such as life‐history strategies or environmental factors. Such species groups include both vertebrates—reptiles (turtles, squamates), fish [Neotropical silversides (*Menidia* spp.), Poeciliids]—and invertebrates (marine worms, parasitic nematodes) (Janzen & Paukstis, 1991; Janzen & Phillips, 2006; Sabath et al., 2016; Tree of Sex Consortium, 2014). For example, turtles have evolved several modes of sex determination including XX/XY and ZZ/ZW sex chromosomes and temperature‐dependent sex determination (TSD) (Bista & Valenzuela, 2020). Indeed, turtles and lizards with sex chromosomes tend to live shorter lives than their TSD counterparts (Sabath et al., 2016). Yet, evidence of turtle senescence is mixed (reviewed in Hoekstra et al., 2020), and the sex specificity of demographic senescence is largely unstudied in turtles (Bronikowski et al., 2021). Whether measures of genome stability (such as DNA repair efficiency, chromosome accessibility, TE dysregulation, epigenetic modification) change in an age‐specific, sex‐specific, or age‐by‐sex‐specific manner in closely related species with variable sex‐determining mechanisms are unknown, yet could provide insights that would help disentangle sex determination from sex‐specific aging.

Sex chromosomes can be lost or newly evolved, even if the sex‐determination system is constant (Furman et al., 2020). Sometimes, sex chromosomes are differentiated, leading to increased transposable element load or heterochromatin levels in one sex but not the other, while in closely related species, the sex chromosomes might show minimal levels of differentiation, as seen in related poecilid fishes (Darolti et al., 2019). Sex chromosomes also differ in size, and, in some species, the sex chromosomes can lead to significant differences in overall genome size between males and females [e.g., in *Drosophila virilis* females have the larger genome, while in *D*. *persimilis*, males have a larger genome (Hjelmen et al., 2019)]. Among species with old sex chromosomes, such as mammals, much of this variation is caused by expansion and rearrangement of ampliconic regions (Brashear et al. 2018; Hughes et al. 2010). Thus, beyond the direct effect of sex chromosomes, genome size, transposable element content, and heterochromatin fraction (and concomitant gene expression) are genomic features that vary between sexes and species and have the potential to impact sex differences in aging by their impact on genome stability. Here again, comparison within and among species of *Drosophila* or fishes with neo‐sex chromosomes, such as sticklebacks (Ross et al., 2009) or African cichlids (Gammerdinger & Kocher, 2018), would allow for the separation of sex‐specific aging from sex chromosomes related to genome stability.

Finally, it is possible to select species to investigate the impact of dosage compensation systems and other sex chromosome regulatory pathways on sex differences in aging. In addition to the genome content, how specific chromosomes, in particular the sex chromosomes, are regulated in males and females can differ significantly. Best known are the dosage compensation systems. These systems, like the dosage compensation complex that upregulates X chromosome genes in *Drosophila melanogaster* males, impact one sex, but not the other. If these sex‐specific regulatory pathways are costly or are likely to break down with age, they might contribute to sex differences in aging. The recent report of environmentally sensitive dosage compensation in turtles with ZZ/ZW sex chromosomes, which is also age‐ and tissue‐dependent (Bista et al. 2021), adds to the complexity of factors potentially affecting sex‐specific aging. Selecting species with similar genomic features that differ in sex chromosome regulation might provide insights into how these pathways impact genome instability and/or sex differences in aging. These comparative studies can then be complemented by work in model species, where either the dosage compensation system or genomic characteristics can be manipulated to test specific hypotheses. Comparative studies across diverse taxa in conjunction with directed experiments in model organisms have the potential to lead to new insights into how dosage compensation systems might impact aging.

### 5.3 Identifying regulatory cascades that control sex differences in aging

Another area of research that we believe could benefit from expanded use of comparative studies concerns the gene regulatory cascades that contribute to aging [e.g., Insulin Insulin‐like Signaling (IIS, p53)] and sex differences in aging (McGaugh et al., 2015; Passow et al., 2019; Tower, 2017). Gene regulatory cascades are often pleiotropic, and currently, it is unknown if the interaction between gene regulatory cascades and sexually dimorphic genomes might contribute to sex differences in aging. For example, the IIS pathway, implicated across animal diversity in regulating aging through nutrient‐sensing and stress responses, interacts with sex‐determination mechanisms (Graze et al., 2018) and alters sexually dimorphic gene expression. As well, signaling through the IIS network can differ between the sexes and the sexes can differ in their responses to treatments that alter IIS signaling (reviewed in Towers, 2017). Even in non‐traditional species, sex differences in IIS gene expression and protein levels have been observed (e.g., Crain et al., 1995; Reding et al., 2016). For example, master sex‐determination genes in vertebrates frequently encode proteins in the TGF‐β (Transforming growth factor beta) signaling pathway (Pan et al., 2021), which regulates many cellular processes (Derynck & Budi, 2019). TGF‐β interacts with the highly evolutionarily conserved IIS/mTor signaling network (e.g., Narasimhan et al., 2011), which underlies trade‐offs between reproduction and survival. It is possible for the sex‐determination pathway to have sex‐specific pleiotropic effects on aging. Another gene set of interest are imprinted genes, as they often impact growth patterns in a sex‐specific manner (Patten et al., 2014). Comparative transcriptome studies and co‐expression networks across species with diverse sex‐determination systems would help to distinguish between pathways that impact aging in both versus just one of the sexes. These studies would also reveal the difference between species‐ or clade‐specific mechanisms and mechanisms that act globally across animal lineages. To reach this level of understanding will likely require the collaboration of scientists from a variety of disciplines, as deep, omics‐level data sets will be needed in addition to a comprehensive understanding of the organisms, their physiology, and life history.

One important feature to consider when investigating regulatory pathways relevant to sex differences in aging is the question of how sexual differentiation is accomplished, that is, via a cell autonomous system or via a hormonal system that affects cells across the body (Bachtrog et al., 2014). In cell autonomous systems (such as seen in birds, *Drosophila* and nematodes), individual cells provide the genetic information to determine their sex, although signals from other cells might have some impact (Murray et al., 2010). In contrast, in cell non‐autonomous, hormonal systems (i.e., in mammals, but see Arnold et al., 2013), specialized cells in the gonad produce sex‐specific hormonal signals that are perceived by the rest of the body and result in a response to this signal, either male or female differentiation. While in species with hormonal sexual differentiation all cells are of the same genotypic and phenotypic sex (precluding somatic mutations), in species with cell autonomous sex determination an individual can be a mosaic of cells with different sexual phenotypes, male, female, or neither. This potential for mosaicism in species with cell autonomous sexual differentiation is seen in gynandromorphs, individuals where a portion of the body shows female characteristics while the rest shows male characteristics. Gynandromorphs have been reported for butterflies, insects, birds, and rodents, which is unexpected given their hormonal sex‐determination system (Major & Smith, 2016; Nakamura, 2009). Gynandromorphs have the potential to provide insights into the complex interactions between cell autonomous and non‐autonomous regulators of sex differences that can occur (e.g., see data from the four core genotypes model in mouse; Arnold & Chen, 2009). Understanding how cells perceive sex and what their identity is will be critical to correctly identifying regulatory pathways that contribute to sex differences in aging.

### 5.4 Taxon selection for comparative studies of sex differences in aging

**FIGURE 4:**
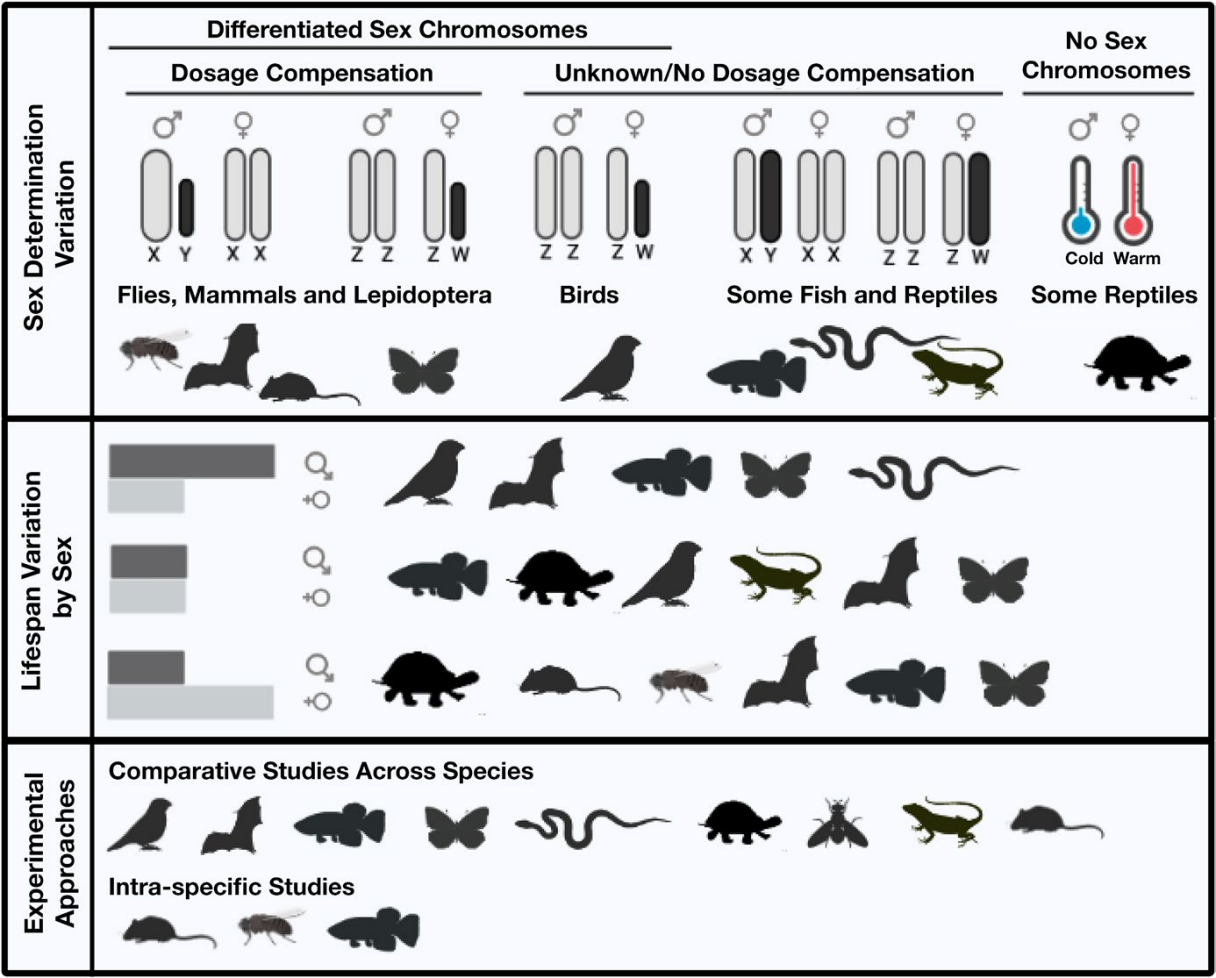
Example taxonomic groups for comparative studies of aging. Species with diverse sex determining mechanisms include those with heterogametic sex chromosomes, non‐differentiated sex chromosomes, and environmental sex determination (warm temperature‐dependent female determination highlighted here; various forms of TSD are found in many reptiles). Species with contrasting patterns of sex‐specific lifespan include species with male‐biased, female‐biased, and unbiased lifespan. And species with inter‐ and intra‐specific variation in aging include diverse wild population and laboratory model species

Species selection will be critical for comparative studies of sex differences in aging. As the examples in earlier paragraphs illustrate, there are many taxa that can provide valuable insights into sex differences in aging (Figure 4). In our opinion, the most promising taxa are those species where most of the factors that are likely to influence aging and sex differences in aging are constant, while ideally only one factor of interest is variable. For example, if our goal is to investigate the role of sex chromosomes, we would choose closely related species from a group, such as the geckos or turtles, where sex chromosomes are present in some species and absent in others. In contrast, if our goal is to investigate the role of size dimorphisms, we would choose species from the same species group, with similar life histories and environments, with one set of species showing sexual size dimorphism, and the other lacking it, with “replication” being provided by multiple independent evolutionary events. Similarly, if we are interested in understanding if warmer climates exacerbate sex differences in aging, we could choose species with wide geographic ranges and compare populations from different parts of the temperature cline. Species that reverse typically seen patterns are also of great interest, such as bird species where the female is larger or more colorful than the male (Amundsen, 2000; Edward & Chapman, 2011). Given the immense variation in both sex differences in aging, as well as in features of interest such as sex‐determination mechanisms, genome size, phenotypic dimorphisms, and more, it is possible to find suitable species groups to investigate a range of hypotheses and include “natural replication” across different taxonomic groups.

Species that exhibit sequential hermaphroditism (individual begins life as one sex, changing to the other sex sometime later in life), are documented in at least 27 families spread across nine teleost orders (Avise & Mank, 2009). Several species of African reed frogs (Grafe & Linsenmair, 1989) would be of great interest, as they could reveal if and how aging trajectories change with a sex change. Ideally, in each case, we would identify two or more species or populations of interest from more than one major branch of the animal tree of life to ensure that what we observe is a general phenomenon rather than a species‐specific oddity. Likely, this approach will require biologists working with model species, lab‐tractable species, captive populations, and wild populations. Despite the inherent challenges in this approach, strategic utilization of the immense variability in both sex differences in aging, as well as the factors hypothesized to control them, is possible and has great potential for the study of sex differences in aging.

### 5.5 Study designs and data types for comparative studies of sex differences in aging

In experimental designs for testing hypotheses of sex‐specific aging, both sexes at various adult ages are needed. However, it can be difficult to define comparable cohorts and samples in diverse animal taxa. Research communities focused on particular taxa typically have an agreed‐upon standard for adults, but these standards often do not translate easily among species groups. For example, many research communities report age as time after sexual maturity, but insect researchers typically report age as time after eclosion. As well, the age of sexual maturity can be different between males and females, which begs the question of whether absolute age versus elapsed age since maturity is the appropriate chronological variable. Similarly, the developmental time prior to hatching can differ between the sexes. Based on our discussions with biologists working with a range of species, there is no simple solution to this problem. However, reporting age according to the species standard (age from eclosion or sexual maturity, etc.), absolute age, and age as a percentage of the maximum lifespan is an approach that allows researchers to compare across species as distinct as insects, fishes, and mammals. For example, defining “young adults” as the first quartile of adult lifespan and “old adults” as the fourth quartile of adult lifespan allows for comparable data sets to be collected from a variety of species (see Ronget & Gaillard, 2020 for additional ideas).

Which tissues to sample are another important question to solve if diverse species are included in a comparative study of sex differences in aging. With studies that span, for example, insects, fishes, and mammals, the task to identify comparable tissues becomes difficult. This issue is further complicated when wild populations are sampled, as the types of tissues that can ethically and efficiently be sampled in a field setting are very limited. Given that data from humans and mice indicate that tissues may have tissue‐specific aging rates (e.g., as in cytosine methylation; Bell et al., 2019; Kling et al., 2020; Zupkovitz et al., 2021), deliberate tissue choice is important. Likely, no “one size fits all” solution is possible, but tissues of interest include muscle, brain, and germ line, as these tissues show clear impacts of senescence in most species. Experimentalists will have to carefully review their choices for which tissues can realistically be sampled and carefully consider their choice in light of the overall study goal.

**FIGURE 5:**
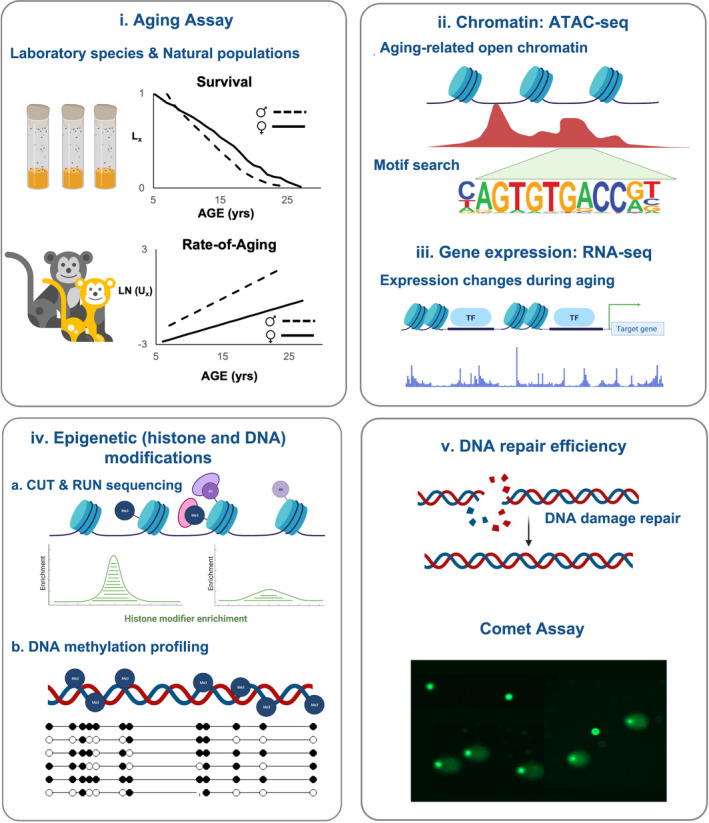
Example study design focused on genome stability changes with advancing age. i) First select a species that has sex‐specific variation in demographic rate‐of‐aging (e.g., females live longer and age slower than males as pictured here for yellow baboons, Bronikowski et al. 2016). ii) Measure age‐related accessible (eu)chromatin and iii) concomitant gene expression. iv) Measure additional age‐related features of the epigenome. v) include functional assays of genome stability such as DNA repair efficiency. These genomic, epigenomic, and functional data can be integrated in deep learning pipelines to develop multi‐variate indices of sex‐and‐age specific change

Finally, the analyses to be conducted on sampled tissues will have to be chosen. Again, data type will depend on the ultimate study goal, but a common minimal set of data and meta‐data might be collected from a large number of species to allow for integration between studies. Meta‐data should include demographic information about the individual sampled (age, sex, tissue, growth conditions, or location for wild species). Age might be difficult to determine, in particular in wild populations, which might need to be considered during the study design. Cytosine methylation clocks provide some promise for determining the age of wild animals that are not part of tagged or monitored populations (for example, see Robeck et al., 2021; Wilkinson et al., 2021). Additional data collected on individuals sampled will depend on the specific question under study, but information about size dimorphisms and overall physical health would be helpful. Finally, molecular measures of aging that can be collected include nuclear and mitochondrial DNA sequences to measure somatic mutations and DNA damage, transcriptomic data to measure aberrant gene expression and activation of transposable elements, telomere length to identify signs of telomere shortening, and chromatin integrity (Figure 5). Additional measures could be DNA damage repair efficiency, levels of stress hormones or antioxidants, the proteome, or metabolites. Limitations on what types of data can be collected will likely depend on the circumstances of collection (field versus laboratory setting), as well as the amount of tissue that can be collected. For example, while 50 mg of tissue—sufficient for transcriptome analysis by RNA‐seq and chromatin integrity analysis by ATAC‐seq (Assay for Transposase‐Accessible Chromatin with high‐throughput sequencing)—can easily be collected from a larger animal, for small animals such as many insects, this tissue amount requires the dissection and pooling of dozens of animals. Similarly, in a laboratory setting samples can be flash frozen immediately to preserve them for metabolomic or proteomic analysis, but in a field setting, tissue preservation methods are often much more limited, and may not be compatible with proteomic or metabolomic analyses. DNA‐based assays are typically compatible with field‐collected samples, but some chromatin and transcriptome assays are possible as well. Given continued development of methods that work with smaller and smaller samples, a promising strategy might be to focus on DNA‐based assays in the short term and to store additional available samples for future investigation of other molecular aspects of aging.

## 6 OUTLOOK

Comparative studies using both intra‐ and inter‐specific experimental designs across the animal kingdom represent a promising opportunity to gain new insights into the origins of sex differences in aging. Technological advances in next‐generation sequencing among other methods have made assays that were until fairly recently restricted to model and laboratory species adaptable to virtually any species of interest. In addition, the amount of tissue needed for these assays has decreased significantly, making it now possible to apply a variety of omics approaches in species across the tree of life. Other assays also have become more sensitive, and non‐invasive alternatives are becoming more available, meaning that more senescence‐related characteristics can now be measured in more species than ever before. The most promising comparative studies will involve biologists from a variety of subdisciplines working together to tackle the question of sex differences in aging from different angles and with complementary approaches. Support from funding agencies and university administrations for these highly interdisciplinary studies will be needed, but the high potential for impact makes these studies worth pursuing.

## CONFLICT OF INTEREST

The authors have no conflict of interest to declare.

## AUTHOR CONTRIBUTIONS

NCR: Conceptualization and Supervision/Project administration. AMB, RPM, PRB, JRW, JEM, EL, GSW, NV, and NCR: Writing—original draft preparation. AMB, RPM, PRB, JRW, JEM, EL, GSW, NV, NCR, AMC, JPdM, J(E)D, AEE, TG, RMG, KEG, JAK: Writing—review and editing. NCR, PRB, J(E)D, JEM: Visualization. NCR, JRW, EL: Funding acquisition.
